# State of the art in childhood nephrotic syndrome: concrete discoveries and unmet needs

**DOI:** 10.3389/fimmu.2023.1167741

**Published:** 2023-07-12

**Authors:** Flavio Vincenti, Andrea Angeletti, Gian Marco Ghiggeri

**Affiliations:** ^1^ Division of Nephrology, Department of Medicine and Department of Surgery, University of California San Francisco, San Francisco, CA, United States; ^2^ Nephrology Dialysis and Transplantation, Istituto di Ricerca e Cura a Carattere Scientifico (IRCCS) Istituto Giannina Gaslini, Genoa, Italy

**Keywords:** nephrotic syndrome, minimal change disease, focal-segmental glomerulosclerosis, post-transplant recurrence, proteinuria, monoclonal antibodies, rituximab

## Abstract

Nephrotic syndrome (NS) is a clinical entity characterized by proteinuria, hypoalbuminemia, and peripheral edema. NS affects about 2–7 per 100,000 children aged below 18 years old yearly and is classified, based on the response to drugs, into steroid sensitive (SSNS), steroid dependent, (SDNS), multidrug dependent (MDNS), and multidrug resistant (MRNS). Forms of NS that are more difficult to treat are associated with a worse outcome with respect to renal function. In particular, MRNS commonly progresses to end stage renal failure requiring renal transplantation, with recurrence of the original disease in half of the cases. Histological presentations of NS may vary from minimal glomerular lesions (MCD) to focal segmental glomerulosclerosis (FSGS) and, of relevance, the histological patterns do not correlate with the response to treatments. Moreover, around half of MRNS cases are secondary to causative pathogenic variants in genes involved in maintaining the glomerular structure. The pathogenesis of NS is still poorly understood and therapeutic approaches are mostly based on clinical experience. Understanding of pathogenetic mechanisms of NS is one of the ‘unmet needs’ in nephrology and represents a significant challenge for the scientific community. The scope of the present review includes exploring relevant findings, identifying unmet needs, and reviewing therapeutic developments that characterize NS in the last decades. The main aim is to provide a basis for new perspectives and mechanistic studies in NS.

## Introduction

Nephrotic syndrome (NS) is a clinical condition that occurs frequently in children and manifests with the classical clinical triad of severe proteinuria, hypoalbuminemia, and diffuse edema (1). Despite homogeneity of the clinical pattern at presentation, NS may evolve with different outcomes, characterized by unpredictable response to drugs and development of renal failure, that probably reflect different pathological entities (1, 2).

Histological patterns of childhood NS vary from minimal glomerular lesions (MCD) to focal areas of segmental sclerosis (FSGS). These two histological opposites share the effacement of the slit diaphragm of podocytes, which effectively sustains the proteinuria (3). If FSGS represents a subsequent stage of MCD or if MCD and FSGS are two distinguished histological entities is still debated (4).

Pathogenesis of childhood NS is largely unknown and, therefore, it is defined as idiopathic NS (iNS) in cases without a definite origin, differentiated from secondary NS characterized by causative mechanisms. An important group of secondary NS has a genetic origin and is characterized by the association with a pathogenic variant in genes transcribing for proteins of podocytes and/or of glomerular structures (5). Causative genetic variants of NS are identified in 66% of congenital and infantile cases, 30% of children and, in approximately 10-15% of young adults presenting with NS (2, 5). A second group of secondary NS occurring in children (but not limited to young ages) is characterized by the temporal associations with either virus infections or drug administration (6, 7).

Therefore, a clear differentiation between iNS and secondary NS has key clinical importance for prognosis and for the choice of therapies since genetic NS has, in general, limited response to drugs (see dedicated part). On the other hand, defying the pathological mechanisms that may sustain iNS and, in particular, iNS resistant to drugs, represents one of the major enigmas in nephrology. Several theories have been developed over the years and the analysis of the most relevant findings in this field will be the focus of the present review.

## How to define idiopathic nephrotic syndrome

As previously reported, childhood NS is a clinical condition characterized by generic signs (i.e. severe proteinuria, hypoalbuminemia, and edema (1)) that may occur in secondary NS and/or in association with several glomerulonephritis common in adults, such as primary and secondary autoimmune forms (ie. membranous and lupus nephropathy), metabolic and genetic conditions (ie. diabetic and hypertensive nephropathy and Alport syndrome), and many others. iNS may be defined on the basis of the above typical symptoms combined with the lack of any evidence of a genetic, infective, inflammatory, or autoimmune cause. Further key factors contributing to the classification of iNS are age at onset, response to treatment, and histological patterns.

### Age

iNS is a disease that typically affects children and young adults (1). Genetic NS, resulting from molecular modifications of podocyte components, usually manifests in the first 12-24 months of life (30% of cases) and has the peak of onset between 2 and 18 years (50-60% of cases); the remaining 10-20% of genetic NS cases present at older ages (2, 5). NS secondary to viral infections and/or associated with drugs may occur any age. Among others, secondary conditions causing NS, such as membranous nephropathy, lupus nephritis, IgA glomerulonephritis, Alport syndrome, metabolic disorders including diabetes mellitus and hypertension, and neoplasms such as myeloma, occur more frequently in adults (8). Therefore, age represents a key classification element of NS.

### Response to drugs

**Figure 1:**
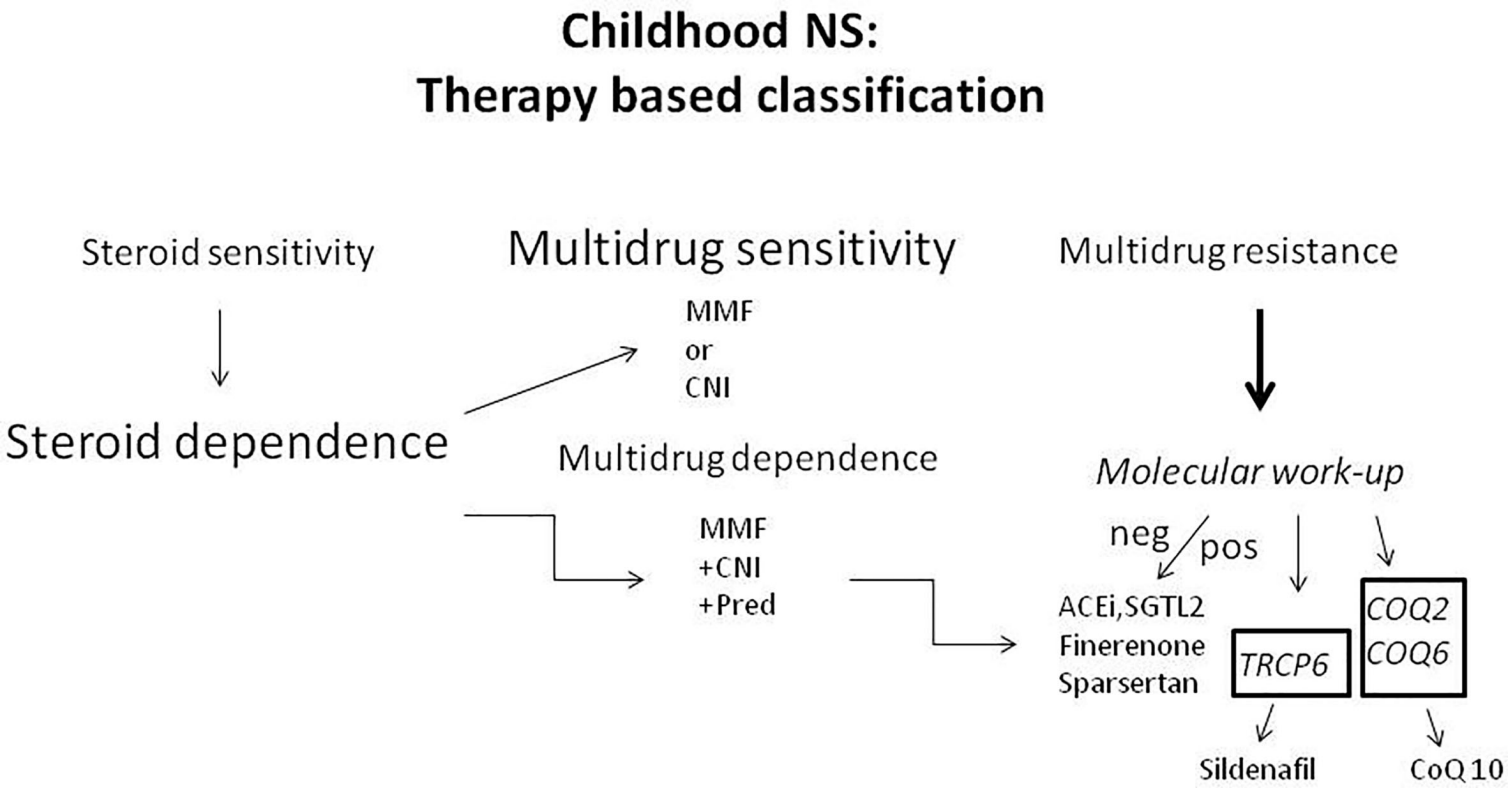
Classification of NS occurring in childhood based on the response to treatments..

The pharmacology approach to iNS (**Figure 1**) has been consolidated over the years and now represents a crucial element for classification. Corticosteroids represent the first step in the treatment of iNS (9, 10). Corticosteroid sensitivity occurs in about 80-90% of new-onset iNS, however, half of them develop corticosteroid dependance (SDNS) and about 10-20% result in corticosteroid resistance (SRNS). Both SDNS and SRNS require the administration of the so=called ‘steroid sparing’ drugs, to reduce the adverse effects correlated with long courses of corticosteroids (11). Classical ‘steroid sparing’ agents are levamisole, mycophenolate mofetyl (MMF), cyclophosphamide, and calcineurin inhibitors (CNI, cyclosporin and tacrolimus) (9, 12). Anti-CD20 monoclonal antibodies have been more recently introduced in therapeutic schemes (13, 14). In SDNS, the administration of a single ‘steroid sparing’ agent is usually effective in maintaining remission, while SRNS may require more than one drug, in which case NS is defined as ‘multidrug dependent’ (MDNS) (15). Limited SRNS that are resistant to the association of any agent and are defined as multidrug resistant (MRNS). These forms account for around 5-10% of overall cases, and no therapeutic approaches are effective in limiting proteinuria. Moreover, in MRNS the progression to renal failure usually occurs in a few years (16, 17).

### Genetic backgrounds

Monogenic NS accounts for a significant number of NS (18). Causative pathogenic gene variants are identified in 66% of congenital and infantile cases, 30% of children and, in approximately 10-15% of young adults presenting with SR/MRNS. So far, more than 70 single gene causes of SRNS/MRNS have been reported (6). NPHS1 (19q13.12) and NPHS2 (1q25.2) are, by far, the two main autosomal recessive genes of genetic NS, while INF2 (14q32.33) and WT1 (11p13) are the leading cause of autosomal dominant NS. See below for the dedicated section (2). Genetic NS must, therefore, be taken into account as a classificatory element in very young patients with NS and in SRNS (see below, the dedicated section).

### Renal pathology

Renal pathology is fundamental to exclude secondary causes of NS, such as autoimmune and inflammatory forms, that occur more frequently in adults. There is consensus in considering a renal pathology approach necessary in NS occurring in adults and in SRNS. In childhood, kidney biopsy is usually not performed at the onset of NS and in subjects with SSNS. KDIGO guidelines suggest limiting kidney biopsy in children to those with a late response to corticosteroids or to those suspicious of an underlying secondary renal pathology (3, 11, 19). Recent findings suggested a possible pathogenetic role of anti-nephrin antibodies in some cases of iNS (20). Based on these findings, the diagnostic approach to iNS consolidated over years may be modified and the indication to kidney biopsy may be revised.

Historically, three major histological patterns have been associated with iNS and still represent useful tools for classification of the disease: a) the absence of any glomerular alteration at the optical microscopy is defined as MCD and transient electron microscopy reveals effacement of podocytes with alteration of the slit diaphragm that is considered the reason for proteinuria; b) the second pattern is more rare and is characterized by mesangial expansion with IgM deposit; and c) the third group presents segmental sclerotic lesions that focally interest glomeruli (FSGS); proliferation of Bowman epithelia and adhesion of glomeruli with the capsule, or tip lesions, are variants of FSGS.

For many years, pathology represented the unique classificatory element of NS until it was proven that FSGS may be the common histological final step of different renal diseases (21). However, the pathological mechanisms explaining the sclerotic degeneration of glomerular tuft common to several glomerulonephritis are still unknown.

The attempt to correlate the different pathology patterns with the response to drugs in children with NS (MCD with SDNS and FSGS with MDNS or MRNS) failed to provide clinical elements for important overlaps between the different categories (5). A still open point is, if FSGS is an evolutionary phase of MCD, this seems in contrast with the reported concept on FSGS as the main feature in many autoimmune and degenerative glomerulonephritis (4).

## Causes of secondary nephrotic syndrome (1): monogenic NS susceptibility

Monogenic NS is the first and most unique case in which either the causes and mechanisms of the disease have been elucidated (**Table 1**). Moreover, genetic susceptibility offers the opportunity to consider mechanisms that predispose to NS where genetics represents a first hit.

**Table 1:** Causes of secondary NS.

Genetic NS.	Ref
Overall	(22–44)
SDNS associated	
GTPase	(27)
MRNS associated	
NPHS1, NPHS2	(29, 35)
ACTN, MYO,INF2	(31, 32, 36)
COQ2,COQ6	(37)
TRCP6	(34)
Susceptibility	
HLA-DQ1, HLA,DQB1	(43)
CALHM6,TNFSF15,Nephrin	(42, 44)
APOL1	(45)
Virus-associated	
SARS-CoV-5 (any age)	(46)
HIV (any age)	(47–52)
Parvovirus (any age)	(53)
Drug-associated	
Pamidronate	(54)
Lithium	(55, 56)
mTOR inhibitors, sirolimus	(57, 58)
VEGF block. Bevacizumab:	(59)
Aflibercet	(59)
tyrosine kinase inhibitor.Ibrutinib	(60, 61)

mTOR, mammalian target of rapamycin; VEGF, vascular endothelial growth factor.

### Monogenic NS

The discovery of genetic backgrounds responsible for mendellian NS has represented a real breakthrough in terms of evolution on pathogenesis and clinical impact (18). To date, almost 70 genes have been described in association with autosomal recessive (prevalent) and dominant (rare) traits of NS including syndromes (22–34) (a detailed review on genes involved in iNS is outside the scope of this review). Most mutations are associated with SRNS; six genes involved in GTPase activity have been recently identified as a cause of SDNS (27). Many genes involved in SRNS code for proteins of the the slit-diaphragm, such as nephrin (*NPHS1*) (35), or are directly linked to this structure, such as podocin (*NPHS2*) (29). Others are components of the cytoskeleton of podocytes such as actinin 4 (*ACTN4*), myosin, and inverted formin 2 (*INF2*) (31, 32, 36). All these molecules are directly involved in maintaining the sieving properties of the glomerulus in terms of selectivity towards the charge and the dimension of circulating proteins. Besides confirming that the podocyte is the basic cell of the glomerulus involved in proteinuria, genetic studies have produced progress in the clinical management since, with the important exceptions of mutations of two mitochondrial genes coding for Coenzymes Q2 (*COQ2*) and Q6 (*COQ6*) (37) and of the transient receptor potential cation channel subfamily C member 6 (TRCP6) (34), genetic NS are only partially responsive to drugs (38) and require only symptomatic and ant-proteinuric generic interventions (**Figure 1**). In the former case, ie. mitochondrial *COQ2* and *COQ6* mutations, oral coenzyme Q10 may revert the clinical phenotypes (37). In the case of the mutation gain of function mutations of *TRCP6*, a Ca^+^ channel important for muscle cells functions, Sildenafil down-regulates the channel expression by reducing the cGMP through a peroxisome proliferator–activated receptor needed for its synthesis (39, 40). Therefore, it was suggested that Sildenafil, reducing the activity of *TRCP6*, could revert this genetic form of NS.

### Susceptibility

The existence of rare familial aggregates of patients with corticosteroid sensitive iNS (41) has long been reported, highlighting the possibility of a common risk linked with susceptibility. The lack of clear inheritance traits supports the existence of a two-hit mechanism, where a susceptibility gene or locus is shared by different individuals in the same family and NS is triggered by a second hit, such as an infectious agent. Familial susceptibility is supported by findings on the genetic architecture of pediatric and adult patients. Genome-wide association studies (GWAS), including a large spectrum of populations in the USA, Europe, and Asia, identified a risk locus in the HLA Class II consisting of *HLA-DQA1* and *HLA-DQB1* and three additional signals, the Calcium Homeostasis Modulator Family Member 6 (*CALHM6*), TNF Superfamily Member 15 (*TNFSF15)*, and nephrin (42–44). The non-HLA loci are linked with expression quantitative trait loci (eQTLs) of monocytes and T cells supporting the concept that an immunomodulatory disfunction has a prominent role in conferring susceptibility to iNS. Large collaborative studies on many thousands of patients is surely the way to obtain further solid information that would rebound on pathogenesis.

*APOL1* is a susceptibility gene for FSGS (45) linked with race that has been documented in subjects of West African ancestry. Adults with this ancestry have, in fact, a 4-5 fold higher risk of developing FSGS than Europeans. Genetic variants of *APOL1* (62, 63) have likely developed in response to (and to protect from) *Trypanosoma brucei rhodesiense* and *Trypanosoma brucei gambiense,* which are very frequent in all African contexts.

## Causes of secondary nephrotic syndrome (2): viruses and drugs

Viruses and Drugs have been proposed as potential causes of secondary NS for patients younger than 18 (**Table 1**)**.** They would be better defined as associated triggers because the real mechanism causing NS is unknown or only partially characterized.

### Viruses

Despite the numeric impact of viral infections associated with NS, the clinical importance is limited since they are potentially reversible (6, 64). Actually, NS has been demonstrated in association with SARS-CoV-5 (in children) and with HIV infections (prevalent in adults).

New episodes of NS were described in two children with acute SARS-CoV-2 infection, whereas the virus has been reported more frequently in association with relapses of NS in patients who had already presented the disease (46). The clinical course has been good in all cases in terms of response to corticosteroids.

HIV infections have been associated with a severe form of collapsing FSGS (HIVAN) complicated by extensive tubular lesions and microcystis (47). In the pre-antiretroviral therapy era, HIVAN represented an important cause of ESRD in many countries even though its incidence has declined with the adoption of specific therapies (48). Studies on mechanisms responsible for HIVAN (49) have shown a direct infection of HIV of podocytes and tubular epithelia leads to the dysregulation of cell cycle with inflammation, alteration of the cytoskeleton, and cell death (50, 51). The high frequency of two variants of Apolipoprotein A1 (G1 and G2) in persons of African ancestry predispose to a very high risk of HIVAN in African Americans and in South Africans with HIV infection (52, 62).

Parvovirus B19 DNA has been isolated in renal tissues of a high percentage of patients with glomerular diseases, including MCD, collapsing FSGS, and membranous nephropathy, suggesting a possible role of the virus in the pathogenesis of the disease (53). The very high frequency of kidney tissues with Parvovirus, absence of specificity, and lack of association with clinical signs of Parvovirus infections speak against a direct involvement in NS.

### Drugs

The administration of drugs has been associated with the development of NS; Pamidronate and Lithium were the first to be reported (54–56). Pamidronate belongs to the class of bisphosphonates that are utilized as first-class agents in osteoporosis and in Paget syndrome. Lithium is a major anti-psychotic drug widely utilized in psychiatric patients. More recently, NS has been reported following treatments with three other categories of drugs, ie. mTOR inhibitors, vascular endothelial growth factor blocking agents, and irreversible Bruton’s tyrosine kinase inhibitors. The mTOR inhibitor sirolimus is an anti-rejection therapy that may substitute other immudepressors in patients with solid organ transplanationis (57); a mechanism for renal toxicity linked with mTOR has been proposed based on the block of podocytes’ compensatory hypertrophy, activated after podocyte loss (58). Also, the intravitreal vascular endothelial growth factor blocking agents bevacizumab and aflibercet are widely utilized in clinical practice, being drugs of choice for macular degeneration and diabetic retinopathy (59). The irreversible Bruton’s tyrosine kinase inhibitor Ibrutinib is essential for the treatment of chronic lymphocytic leukemia (65) and mantle-cell lymphoma (66). Several reports describe the occurrence of NS following Ibrutinib, but the mechanisms are not understood (60, 61).

## Mechanisms for idiopathic nephrotic syndrome (1): membrane selectivity, oxidative stress, and circulating factors

**Figure 2:**
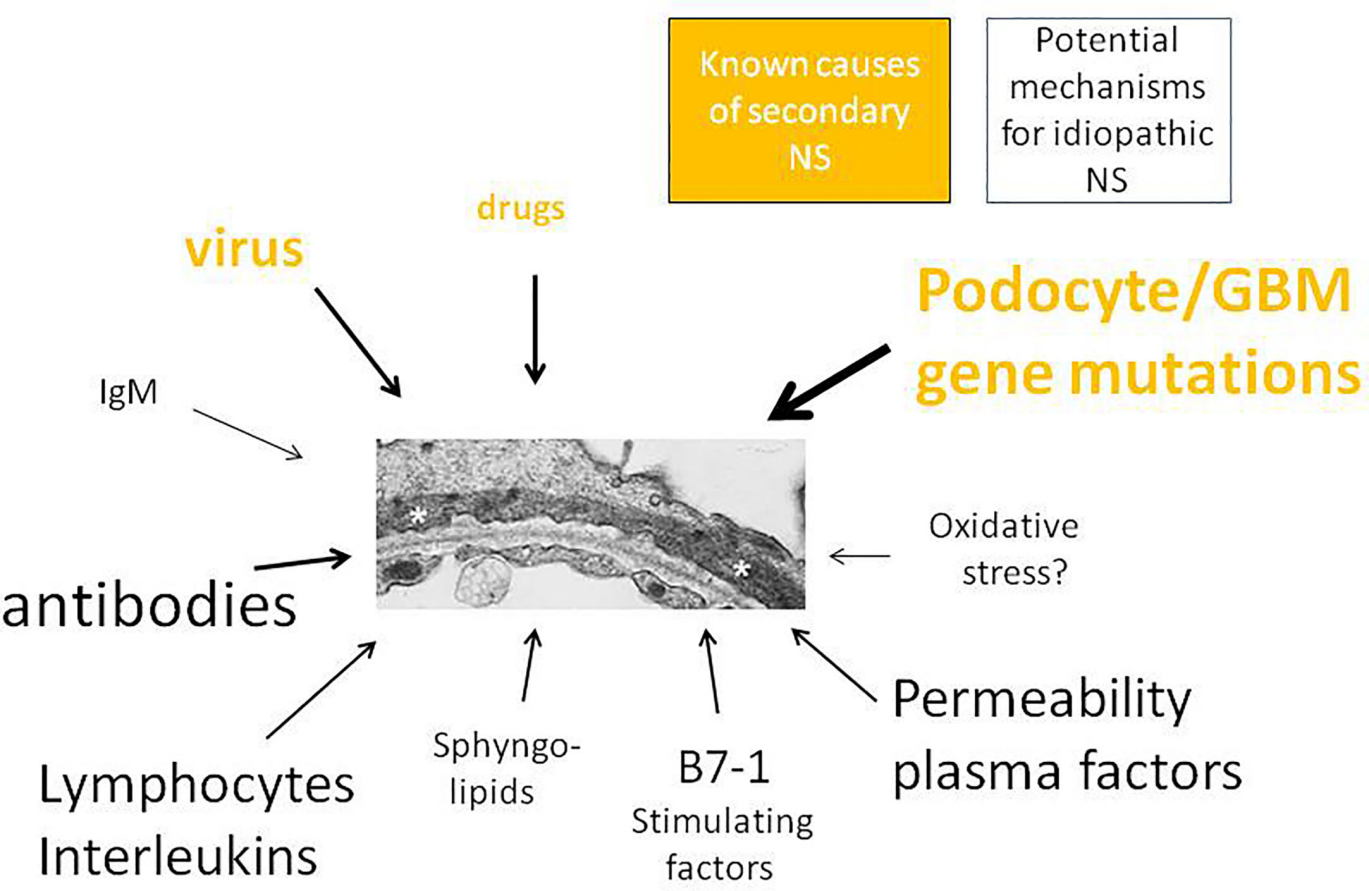
Primary and secondary childhood NS. Inherited forms of NS, caused by pathological variants in genes transcribing for the main protein of glomerular tuft, represent a unique group of NS for which a clear mechanism has been defined. NS associated with either virus infections or drugs are reported as secondary NS on the basis of both the temporal association and reversibility of proteinuria. All other mechanisms are supposed and require further confirmatory results..

The sections below are dedicated to those forms of NS that usually occur in children without a recognized cause and/or of pathogenetic mechanisms. Therefore, as previously mentioned, these forms are defined as iNS. We will summarize the most relevant pathological theories proposed over the years. Despite the absence of any definitive demonstration, the proposed theories may still represent potential future lines of research to be developed and explored (**Figure 2** **;** **Table 2**).

**Table 2:** Idiopathic Nephrotic syndrome (iNS).

Circulating Plasma Factors/ Two hit mechanisms	References
CD40L	(67–78)
c-mip	(79–84)
uPA/suPAR	(85–87)
Angiopoietin-like 4	(88–91)
Hemopexin	(92–96)
Cationic albumin	(97–99)
B7-1 (CD80)	(78, 100–106)
Antibodies/ Regulatory Immunoglobulins	
Anti-nephrin	(20)
Anti-CD40	(75)
Anti-actin/ATP synthase Anti- UCHL1 Delville’s group Sialylated IgM	(107) (108) (109) (110)
Oxidative stress	
α1-antitrypsin	(111, 112)
Glomerular Basement Membrane	
Sialic acid, Sphingolipids	(113, 114)
T cells/Interleukins	
IL1/IL1R	(115, 116)
Th2/IL4, IL5, IL9, IL10, IL13	(117–126)
Th17/IL17	(127)
Regulatory T cells	(124, 128–132)
B cell subsets	
Switched B cells	(133)

This table covers a wide range of potential factors implicated in the pathogenesis of iNS that should be further confirmed.

### Glomerular barrier sieving property modification

The glomerular basement membrane (GBM) is a selective filter that allows the passage of proteins according to size and electrical charge: molecules between 10 and 36 Armstrong (A) are filtered in relation to size (maximal for 10 A) whereas molecules > 36 A are filtered on the basis of the charge with privilege for those with a cationic isoelectric point (pI>7) (134, 135). This function is mostly due to the presence of sialic aid in the external membrane of podocytes that confers a negative charge to the structure. In accordance with the function of sialic acid, podocyte-specific ablation of sialylation in mice caused a phenotype resembling human FSGS (113, 136). Also, sphingolipids play a crucial role in maintaining renal permeability. In line, previous reports suggest that their accumulation in podocytes characterize several metabolic and non-metabolic diseases, including FSGS, and may contribute to altering glomerular permeability (114, 137).

In normal conditions, urine contains minimal amounts of proteins since cationic proteins are in trace amounts in serum and small proteins that cross the glomerular filter are reabsorbed by proximal tubular cells. Filtered albumin is also almost completely reabsorbed by proximal tubular cells *via* endocytosis (138, 139).

iNS is caused by an alteration of the podocyte filtering unit wherein the capacity to repulse proteins on the basis of size and charge is only partially maintained and anionic proteins of any size can pass the filter and are lost into urine. Studies carried out between 1970 and 1980 showed that the selectivity index calculated from urinary excretion and tubular reabsorption (determined by the direct staining in renal biopsies) of albumin and IgG correlated with the intensity of pathological lesions (140), supporting the concomitant reduction in both pore size and density of GBM in MCD (97).

However, modifications of the charge of circulating proteins may partially sustain proteinuria and be a further pathogenetic factor in NS. Studies on charge selectivity were performed considering albumin as a model of sieving modifications, since it accounts for 90% of all urinary proteins in MCD. Albumin is a 46A molecule with anionic charge (pI 4.6). In normal conditions, it is completely repulsed by the podocyte filtering unit for either size and charge characteristics. In MCD, about 50% of urinary albumin isoforms are more cationic compared to serum albumin (4.8-5.2 *vs*. 4.6) and are also more cationic compared to urinary albumin after remission of the disease. In FSGS, urinary and serum albumin have the same charge, suggesting that charge selectivity of albumin is partially maintained in MCD whereas it is lost in FSGS (98).

Other studies reported that higher cationic isoforms of albumin are characterized by lower amounts of fatty acids compared to the anionic compounds (99). This finding makes sense, since fatty acids that are transported by albumin in serum confer the characteristic anionic charge to this protein. However, other anionic residues may be implicated in regulating charge properties of circulating proteins. Among others, sialic acid is a strong acidic molecule (pK 2.6-2.9) that is transported in serum by a few proteins such as immunoglobulins, fibrinogen, and alpha-2-macrogobulin. Sialidase enzymes regulate the sialylation of transporter proteins and, in parallel, their charge is anionic for sialylated proteins and cationic for de-sialylated isoforms. Recent findings demonstrate that sialylation of immunoglobulins modulate their cell binding and play an important role in determining a pathological function (141, 142). One example is rheumatoid arthritis, where the ratio between de-sialylated and sialylated immunoglobulins directly correlated with the activity of the disease (143–145). Moreover, variations in sialic acid of immunoglobulins, and in particular of IgM, modulate the immunoinflammatory functions of T cells in MCD (110). Therefore, more extensive analysis to investigate the possible pathological role of IgM sialydation in iNS is necessary.

### Oxidative stress

iNS is commonly associated with a systemic oxidative milieu. Extensive proteolysis of albumin and α1-antitrypsin has been documented in the plasma and urine of patients with FSGS (111, 112). α1-antitrypsin is an enzyme with anti-proteolytic activity whose fragmentation implies increased proteolysis. Albumin is the principal anti-oxidant in blood: the SH residue of Cys34 is sulphonated in the presence of an oxidative stress with the addition of ±48Da molecular weight and changes of the net charge for the addition of negative residues (146, 147). The increased amounts of oxidized albumin in FSGS demonstrates the oxidative stress. Other indirect evidence supports the implication of oxidants in FSGS. Patients carrying a mutation in the two mitochondrial genes *COQ2* and *COQ6* develop FSGS and revert the phenotype after supplementation with Coenzyme Q10 (37). All experimental models of FSGS utilize oxidative substances such as adriamycin and puromicin (148, 149). Overall, there is a direct correlation between oxidation and proteolysis that is able to damage renal structures.

### Circulating permeability plasma factors

iNS and, in particular, FSGS have been considered the results of circulating plasma factors that bind and permeabilize the filtering glomerular unit (67, 68, 150). Recurrence of NS in the transplanted graft is often used as evidence of the existence of a circulating permeability factor that mediates glomerular injury in around 50% of patients (69–71). The experience described by Gallon et al. (72)represents definitive support of this concept. They removed a renal graft after a few days of transplantation from a subject with FSGS presenting recurrence of the disease immediately after the kidney transplant. The graft was then transplanted in a second subject with ESRF due to a malformative renal disease, observing the recovery of podocyte effacement previously demonstrated by the renal biopsy during the first transplant and absence of proteinuria in the second patient.

The story of pathogenesis of FSGS coincided for many years with the research of permeability circulating plasma factors. Initial studies tested the efficacy of several potential molecules in inducing permeability in an *in vitro* model with isolated glomeruli (73). A few molecules emerged as potential plasma factors, however, we are still far from any definitive conclusion and the nature of the permeability factor is still pending (67). We here describe some molecules that have been reported as valuable candidates for permeability factors.

#### CD40L

The interest on CD40L and its natural target CD40 derives from the experimental observation that CD40L promotes redistribution of nephrin in both podocytes and glomeruli and increases permeability to albumin (74, 75). CD40L exists as a soluble circulating factor and CD40 is a costimulatory molecule present on the surface of cells involved in immunologic response such as B cells and monocytes/macrophages. CD40 is also constitutively expressed in podocytes (75). CD40L/CD40 axis promotes inflammatory events (76, 77) with activation of metalloproteases, chemokines, urokinase, and the soluble urokinase plasminogen activator receptor (suPAR). Serum levels of sCD40L are increased in children with both SSNS and SRNS and in adult patients with biopsy-proven FSGS compared to healthy subjects (75). On the other hand, serum levels of CD40L are comparable in children with congenital NS and in patients with membranous nephropathy.

*In vivo* studies have shown that CD40 is strongly expressed in podocytes of patients with both primary and post-transplant recurrent FSGS and the presence of circulating anti-CD40 antibodies are present in the serum of the same patients (78). It has been proposed that anti-CD40 antibodies purified from the serum of patients with recurrent FSGS disrupt the podocyte structure by acting on F-actin filaments with the involvement of the suPAR–β3 integrin signaling pathway (78).

#### c-Mip

c-Mip (C-maf inducing protein) is an 86 KDa protein with unclear functions that is upregulated in the podocytes of patients with NS in association with conditions such as cancer that predispose the development of NS. c-Mip levels also increase in concomitance with drugs, such as the angiogenesis inhibitors sorafenib and sunitinib (79–81) that may associate with NS. *In vivo* studies showed that transgenic mice over-expressing c-Mip develop a disease resembling human MCD (82). However, data on c-Mip serum levels in patients with NS are not available and no pathological mechanisms to correlate c-Mip and proteinuria have been proposed. In the case of inhibitors of angiogenesis that act on vascular endothelial growth factor-receptor tyrosine kinase (VEGF-TKIs), it has been hypothesized that the reduction of RelA, a member of the NFk-B family, is an inhibitor of c-Mip (80). More generally, one explanation for proteinuria occurring in association with c-Mip stimulation is that it interacts with factors such as Fyn, a Src kinase that is involved in nephrin phosphorylation (83) and, therefore, c-Mip may directly affect the podocyte slit diaphragm (84).

#### uPA/suPAR

The possible implication of suPAR in FSGS derives from *in vivo* animal models of the disease and does not reflect observation in humans (85, 86). In fact, serum levels of suPAR are not increased in NS and simply correlate with renal function (87). This molecule is a determinant of proteinuria in experimental models of FSGS; mice lacking uPAR (*PLAUR*-/-) are protected from developing proteinuria (85). The plasminogen activator receptor (uPAR) is expressed by podocytes and it functions to maintain podocyte shape and sieve properties by modifying the αvβ3-integrin assembly and adhesion to extracellular matrix.

#### Angiopoietin-like 4

ANGPTL4 is a glycoprotein expressed by several tissues and organs, including glomeruli. It is a known inhibitor of lipoprotein lipase (an enzyme that catalyzes conversion of triglycerides to monoglycerides and free fatty acids) and plays a main role in reducing triglyceride levels in circulation. ANGPTL4 has two isoforms with elevated (pI >8) and neutral (pI 7) isoelectric point (88) that are characterized by different sialic acid contents: sialylated ANGPTL4 (the neutral isoform found in circulation) is secreted in peripheral organs (mostly skeletal muscle, heart, and adipose tissue) while the hypo-sialylated isoform is produced by podocyte and remains restricted to the kidney. Several observations indicate a potential involvement of ANGPTL4 in NS, but the interpretation of data is not univocal. In fact, circulating sislylated ANGPTL4 is high in NS and plays an anti-proteinuric effect by binding αvβ5 integrin in glomerular endhotelim (89) whereas the renal hypo-syalilated isoform determines proteinuria (90). In experimental models of NS, levels of the two isoforms of ANGPTL4 are under the control, with an opposite effect, of corticosteroids that up-regulate the expression of sialylated isoforms in adipose tissue and increase their circulating levels while reducing ANGPTL4 expression in podocytes (91). Therefore, the efficacy of corticosteroids in human NS is likely linked with the combined ability to reduce a potential mediator of proteinuria (renal ANGPTL4) and increase circulating levels of ANGPTL4 that plays an anti-proteinuric effect.

#### Hemopexin

Plasma hemopexin is a glycoprotein with serine protease activity (92) involved in iron homeostasis as a binder of free heme. It is considered an antioxidant protein, mostly expressed during acute phases of inflammation (93, 94). Rats treated with a tail infusion of hemopexin developed reversible proteinuria (95). In humans, plasma and urine levels of hemopexin have been reported to be decreased in subjects with acute relapse of MCD, compared to the high levels described in proteinuric subjects with FSGS, membranous proliferative glomerulonephritis, or IgA nephropathy, suggesting specificity of changes for MCD (96).

### Second-hit mechanism (B7-1)

B7-1 (CD80) is a costimulatory ligand expressed on the surface of antigen-presenting cells (APCs), the binding of which to the T- cell receptors CD28 and CTLA-4 is essential for activating and regulating T-cell immunity. Previous findings described the B7 expression on podocytes’ surface of subjects with NS and in post-transplant recurrence of FSGS (78, 100, 101). The majority of studies favor the idea that B7-1 may represent a non-specific marker of proteinuric disease, such as MCD, lupus nephritis, membranous nephropathy, and diabetic nephropathy, but not in FSGS (102–105). Therefore, *de novo* expression of glomerular B7-1 in FSGS recurrence may be considered as a local tissue response to non-specific stimuli (ie. oxidants, inflammatory, infectious) (106). The expression of B7-1 secondary to these stimuli represent a possible antigen, as part of a second-hit mechanism. Such findings suggest caution in interpretating the significance of glomerular B7-1 expression in post-transplant FSGS recurrence. The reason why B7-1 expression is expressed only in limited patients with post-transplant recurrence of FSGS is unclear. However, such reports raised the hypothesis that B7 blockade may result in a podocyte-protective effect with consequent reduction of proteinuria. Abatacept, a B7-1 inhibitor, was proposed as a specific therapeutic agent in post-transplant FSGS recurrence, with discordant results. In the most recent study, Burke III et al. (101 *)*, proposed the administration of abatacept, based on the B7-1 podocytes expression at kidney biopsy, in 12 subjects (median age 12 years old) with NS recurrence after kidney transplant and resistant to conventional treatments with plasmapheresis and rituximab. Nine subjects responded to treatment, of whom seven had a kidney biopsy positive for B7-1, while two were without biopsy. Of note, of the three patients not responding to abatacept, one had a kidney biopsy positive for B7-1. Based on these results, authors suggested that B7-1 podocyte staining may identify subjects who can benefit from abatacept.

## Mechanisms for idiopathic nephrotic syndrome (2): complement, interleukines, and immune cells

### C3a/C3aR-IL1β loop

Recent findings suggested that regulation of C3 convertase in podocytes by CD55, a decay-accelerating factor, is implicated in determining proteinuria and glomerular sclerosis in mice models of glomerulosclerosis induced by adriamycin (115). C3a/C3aR interaction enhances inflammasome activation in podocytes and the release of active IL-1β that binds IL-1R1 and leads to actin cytoskeleton rearrangement and podocyte disarrangement. Proteinuria in these mice prevents the uncoupling of IL-1β/IL-1R1 signaling, providing a causal link (115). IL-1β is a member of the interleukin 1 family of cytokines involved in the inflammatory response that is produced by macrophages, monocytes, and dendritic cells.

Anakinra, the receptor antagonist binding IL-1 β to IL-1R1, represents the treatment of choice in several rheumatic diseases. Based on the *in vitro* and *in vivo* findings, Anegelli and co (116). recently administered Anakinra in two patients with multidrug-dependent/-resistant NS and in one patient with post-transplant recurrence of FSGS. They induced a complete and two partial remissions respectively (116). The limited findings supported the hypothesis that, in proteinuric disease, C3a acts as a second-hit in mediating podocyte cytoskeleton rearrangement and that IL-1R1 blockers may limit the damage (116).

### T cells/Interleukins

A generic role of T cells was supported by the observation that supernatants of hybridomes from patients with MCD efface podocytes and induce proteinuria in rats (117). This finding reinforced the concept that MCD is caused by T-cell disorder (118) and stimulated many studies aimed to characterize the T cell compartment in iNS, without definitive results. Observational studies documented that concomitant measle infections, which stimulate a Tcell response, are associated with proteinuria reduction in iNS patients (119). Similar associations were reported during type B influenza (120) and ZIKA virus infections (121).

#### T helper 2 cells (Th2)

iNS, in particular MCD, is usually characterized by an increased serum level of several cytokines (IL-4, IL-5, IL-9, IL-10, and IL-13) that may be indirectly related to the activation of T helper 2 cells (122, 123). IL13 mRNA is up-regulated in the renal tissue of children with MCD and is associated with increased release of IL13 by CD3^+^ T cells (122). Moreover, *in vivo* experiments support a possible role for IL-13 in iNS. Wistar rats over-expressing IL13 spontaneously develop proteinuria and podocyte effacement, while Minnesota-Buffalo, a strain of rats that spontaneously develop proteinuria, have high levels of IL4 and IL13 that precede proteinuria (124). Anti-IL13 drugs are now available for human administration and have already been reported as effective in the treatment of subjects with asthma (125) and atopic dermatitis (126). Given the safety of the drug, a small pilot study testing the effectiveness of anti-IL-13 in MCD would be of interest.

#### T helper 17 cells (Th17)

IL-17 is produced by T helper 17 cells (Th17) that derive from naïve CD4^+^ after stimulation from IL-6 and IL-13. Previous reports associated high serum levels of IL-17 with glomerulosclerosis in patients with FSGS (127).

#### Regulatory T cells (Tregs)

Tregs (CD4+ CD25+ Foxp3+) are a subpopulation of T cells, stimulated by IL-2, that suppress immune response. Tregs are able to inhibit T cell proliferation and cytokine production and play a critical role in preventing autoimmunity. Previous papers reported that circulating Tregs are reduced in children affected by MCD and that proteinuria may be reverted by the infusion of Tregs (124, 128) in several murine models of NS (ie. adriamycin, Buffalo-Mna, and LPS). Moreover, patients affected by IPEX, an immunodeficiency hereditary syndrome with polyendocrinopathy and enteropathy characterized by reduced circulating levels of Tregs due to Foxp3 inactivation, may develop MCD (129). Of interest, the reconstitution of the entire T cell compartment after bone marrow transplantation in children with IPEX recover the general symptoms of the syndrome and also normalize the glomerular disease (130). Tregs are stimulated and increased by IL-2 (131, 132). While the data above support a protective effect of Tregs in NS, the unique report in children with NS treated with IL2 contradicted this concept. In fact, Bonanni et al. (151) infused IL2 in five children affected by SRNS who presented lower circulating level of Tregs compared to healthy subjects and obtained the normalization of circulating Tregs without any effect on proteinuria.

### B cells

For decades, NS has been considered a T cell pathology based on the efficacy of steroids and on the absence of antibody deposition in glomeruli. Results obtained with anti-CD20 monoclonal antibodies have modified this belief. Starting from 2010, several randomized studies proved the non-inferiority of the chimeric anti-CD20 antibody rituximab compared to steroids and CNI in the treatment of steroid-dependent and resistant NS (13, 14, 16, 152–154). Such clinical results strongly support a possible implication of B cells in this pathology. It was initially proved that the positive effect of rituximab is correlated with the depletory effect on B cells but successive studies have widened the panel of cells expressing CD20 to B memory cells, which are now considered the cell effector of rituximab in terms of length of relapse (155). Through time-of-flight mass cytometry (CyTOF), it was observed that frequencies of class-switched B cells were significantly higher in patients who received rituximab and relapsed than in non-relapsing individuals (133), a phenomenon already observed in autoimmune conditions such as myasthenia gravis, neuromyelitis optica, and rheumatoid arthritis (156, 157). The phenotype frequently associated with recurrence of NS is IgDCD27+CD38+CD95+ Ab-secreting where the presence of CD38 promotes survival in germinal center B cells. The meaning of B cells and, in particular, of switched B cells expressing CD38 in NS can be interpreted as the persistent possibility of secreting antibodies and should be connected with a potential role of antibodies and, more in general, of immunoglobulins. More observational and experimental evidence of an antibody role in iNS is needed since, with the exception of anti-nephrin antibodies described in MCD (20), NS patients do not present immune-deposits in glomeruli. This is an evolving story that started only recently and needs to be developed in detail (see the section dedicated to antibodies below).

## Proposed mechanisms for idiopathic nephrotic syndrome (3): antibodies and regulatory immunoglobulins

### Antibodies

That auto-antibodies may have a pathological role in iNS is not a novel thought at all. However, the possible pathological relevance of auto-antibodies was further considered mostly after the introduction of treatments with anti-CD20 monoclonal antibodies. In previous literature, several papers proposed many possible circulating antibodies as responsible for the disease (see previous section).

Among others, Musante et al. (107) reported that circulating anti-actin/ATP synthase beta chain IgM were present in the serum of around 10% of FSGS patients. Moreover, authors described that infusion of such antibodies in Sprague Dawley rats induced proteinuria, which correlated with IgM glomerular deposition. In 2014, Delville et al. (109) proposed a selection of circulating antibodies with several antigens as targets as biomarkers of post-transplant recurrence FSGS: protein tyrosine phosphatase receptor O, TNF receptor superfamily member 6, Chorionic gonadotropin β, Ribonucleoprotein B, Apoliprotein 2, P2Y purinoceptor 11, Retinoid orphan nuclear receptor α, Chemokine C-C motif-ligand 19, Myosin light kinase, and CD40 (109). Antibody anti-CD40 was deeply investigated. *In vitro* studies showed that podocytes express CD40 and that treatment with antibody anti-CD40 resulted in morphologic alterations. Moreover, antibody anti-CD40 induced proteinuria when administered in wild type mice (75). More recently, Jamin et al. (158) identified anti-Ubiquitin Carboxyl-Terminal Hydrolase L1 (UCHL1) IgG in patients with relapsing proteinuria and Ye et al. (108) described anti-Annexin A2 antibodies in patients with NS with IgG deposits along with GBM. The most recent study by Watts et al. (20) described the presence of circulating anti-nephrin IgG in patients with MCD and also demonstrated their binding to podocytes’ cytoplasm and co-localization with nephrin. Moreover, serum levels of circulating anti-nephrin antibodies correlated with proteinuria and disappeared after remission, suggesting a direct pathogenetic implication.

Overall, available evidence suggests the existence of circulating anti-podocyte antibodies that may characterize different subsets of NS. However, with the exception of anti-nephrin IgG, other studies did not show the concomitance of circulating antibodies with their deposition in glomeruli.

Furthermore, the limited efficacy of anti-CD20 monoclonal antibodies in inducing complete remission, in particular in SRNS, suggests the need for caution and requires more evidence on the pathological role of such antibodies.

### Regulatory immunoglobulins

Immunoglobulins may play an important role in NS, with pathological mechanisms different from the classical immune deposits of glomerulonephritis. Previous studies demonstrated that IgM may deposit on the surface of T cells. After deposition, sialylated IgM are internalized differently to non-sialylated IgM, which remain on surface. The internalization of sialylated IgM correlate with T cells proliferation and confer more resistance to steroids (141). Moreover, internalization of IgM and T cells’ proliferation are both associated with relapse of proteinuria in patients with SDNS (110). Rituximab seems to have more effect in decreasing the sialylated IgM; therefore we may speculate that the efficacy of rituximab in SSNS may be partially explained by selective depletion of sialylated IgM on T cells.

## Therapies for nephrotic syndrome

A detailed analysis and comparison of therapies administered in NS is outside the scope of this review. However, we here present an overview of the therapeutical progresses of the last two decades, with the aim to define how the pathological progresses have been accompanied by therapeutical ones.

### Corticosteroids

Steroids still represent the treatment of reference for iNS in case of occurrence and relapses. Based on the KDIGO Guidelines (159), prednisone at a dose of 60 mg/m (2) for 6 weeks following 6 weeks at 40 mg/m (2) is associated with the best outcome in terms of early relapses and lower incidence of development of SDNS. The administration of different type of corticosteroids, such as methyl-prednisolone or deflazacort, is not associated with better outcome compared to the scheme reported above. Relapse of iNS is treated with prednisone 40 mg/m (2) every day until normalization of proteinuria and then the same dose given every other day for 40 days. Longer schemes do not improve the outcome in terms of relapse of proteinuria.

### Steroid-sparing agents

**L**ong-term complications related to the chronic administration of steroids in SDNS are commonly reported. Therefore, steroid-sparing agents (levamisole, cyclophosphamide, alkylating agents, calcinerin inhibitors, mycophenolate mofetil, and rituximab) are administered with the aim to limit the overall steroid dose (17, 160). Previous randomized clinical studies showed similar efficacy among the different steroid-sparing agents (13, 14, 152, 153, 161, 162). Therefore, the choice may depend on the evaluation of possible side effects and safety (126, 127) and on practical aspects. As an example, a single infusion of rituximab allows to avoid the daily administration of therapy. However, several concerns still remain open on rituximab, such as the missing definition of a definitive cumulative dose: recent studies compared the efficacy of different rituximab courses, demonstrating that SDNS patients receiving rituximab for the third or fourth episode of NS relapse had longer remission than patients receiving rituximab for the first release (163). Moreover, circulating levels of memory B cells better correlate with NS remission (155) than total B cells and may represent a potential marker for defining the needing of further rituximab infusion.

Also, the development of anti-rituximab antibodies, due to the chimeric nature of these antibodies, was proposed as a factor limiting the efficacy of rituximab (164). In our experience, in a significant cohort of NS patients who received at least two rituximab infusions, the development of circulating anti-rituximab antibodies did not affect the response to the treatment (165).

### Multidrug dependence

A subset of patients with NS develop multidrug dependence (MDNS), defined as the need of more than one drug, usually calcinerin inhibitors and micophenolate mofetyl, to keep remission. MDNS is characterized by frequent relapses of NS and may evolve to MRNS that in extreme conditions, however, a specific therapeutical approach for these patients has not yet been proposed.

### Multidrug resistance

Currently, the treatment of patients affected by multidrug resistance mostly consist of anti-proteinuric therapies, such as mineral corticoid antagonists (finrenone) and/or drugs that influence the angiotensin and the endothelin renal accomodation (166, 167). The main aim of such conservative therapies is to limit the progression to end-stage renal disease. Therefore, MRNS represents one of the most relevant unmet needs in nephrology. A second generation of humanized anti-CD20 monoclonal antibodies, such as ofatumumab (154) or obinutuzumab (168), or therapeutical schemes based on the association of different biologics, have been recently proposed (169).

## Conclusions

### Concrete discoveries

In the last two decades, in the context of childhood NS, the main findings were represented by the definition of a genetic basis of mendelian forms and by the demonstrated efficacy of anti-CD20 monoclonal antibodies in SDNS.

When monogenic NS is promptly diagnosed, the therapeutical approach requires the administration of antiproteinuric treatments, limiting therefore the immunosuppressive drugs with relative adverse events. Very recently, cyclosporin was demonstrated to be effective in reducing proteinuria in selected patients with monogenic NS (38).

Anti-CD20 monoclonal antibodies have modified the therapeutical approach to SDNS with some advantages over previous drugs. An important consequence of this finding is the strong suggestion that B cells may be implicated in iNS. Such findings provided the bases for new studies on the characterization of B cell subsets in iNS.

### Urgent unmet needs

The most urgent unmet need is the development of effective therapies in MRNS and in post-transplant NS recurrence. The definition of the pathological mechanisms causing both drug sensitive and resistant iNS remain fundamental and should precede any therapeutic innovation. The efficacy of anti-CD20 has stimulated the research on antibodies and on regulatory immunoglobulins in iNS. Previous data highlighted the existence of circulating IgM with variable charge and isoforms with cationic pI. Whether cationic IgM play a role in iNS and how they are modified by anti-CD20 drugs is a matter of research. Another point of interest is the need to better characterize the amount of immunoglobulins that escape the anti-CD20 effect. New anti-CD20 monoclonal antibodies may have more efficacy in the treatment of MRNS and post-transplant recurrence NS.

The association of anti-CD20 with other monoclonal antibodies targeting long-lived cells producing antibodies, such as plasma cells that expressed CD38, represent the proof-of-concept for an ongoing Phase II study in MDNS, MRNS, and post-transplant recurrence NS (NCT05704400).

## Author contributions

All authors listed have made a substantial, direct, and intellectual contribution to the work and approved it for publication.
