# Supplementary figures and images for: State of the art in childhood nephrotic syndrome: concrete discoveries and unmet needs
Source: Front Immunol. 2023 Jul 12;14:1167741. doi: 10.3389/fimmu.2023.1167741 (PMC10368981; doi:10.3389/fimmu.2023.1167741)

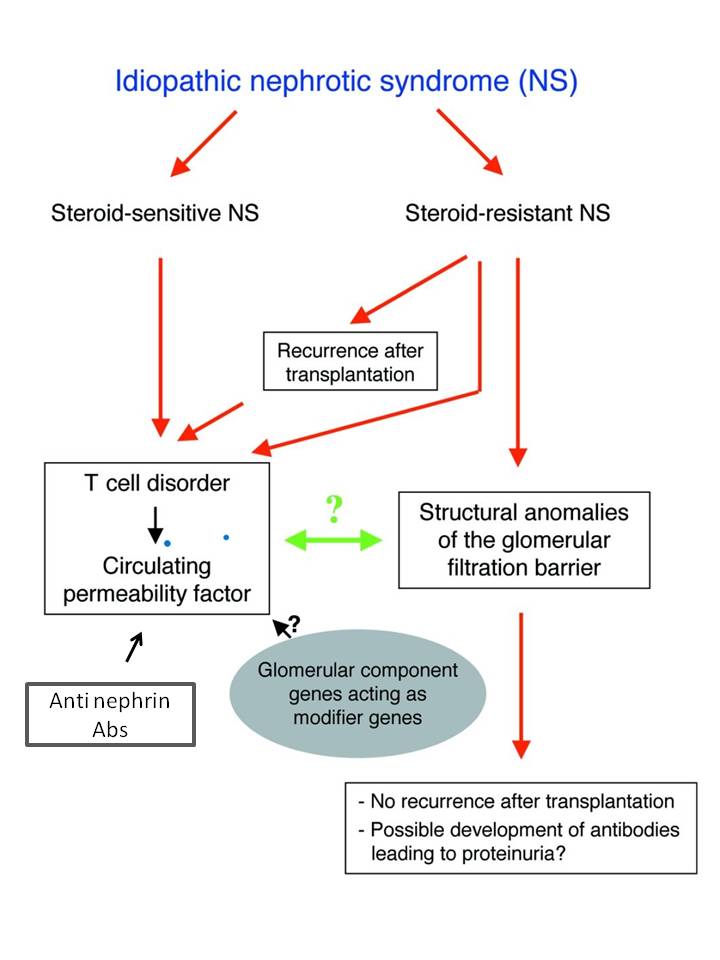

Supplement: Supplementary file 1 [file Image_1.jpeg]
